# Draft genome sequence of *Prototheca bovis* strain P18 isolated from cattle in Japan

**DOI:** 10.1128/mra.00544-25

**Published:** 2025-07-31

**Authors:** Masato Akiba, Tsuyoshi Sekizuka, Ichiro Yasutomi, Yuichiro Fujii, Ikuo Uchida

**Affiliations:** 1 School of Veterinary Medicine, Rakuno Gakuen University 13022, Ebetsu, Hokkaido, Japan; 2 SEKIZUKA Industry Co., Ltd., Ome, Tokyo, Japan; 3 Yubetsu Herd Management Service, Yubetsu, Hokkaido, Japan; 4 Fujii Bokujo, Furano, Hokkaido, Japan; University of California Riverside, Riverside, California, USA

**Keywords:** *Prototheca bovis*, algae, cattle, mastitis

## Abstract

*Prototheca bovis* is a causative agent of bovine mastitis, which is often refractory to treatment and can lead to substantial economic losses in the dairy industry worldwide. This study presents the draft genome sequence of strain P18 isolated in Japan as a reference for Japanese *P. bovis* isolates.

## ANNOUNCEMENT

The genus *Prototheca* (Trebouxiophyceae, Chlorophyta) comprises unicellular, nonphotosynthetic, and saprophytic microalgae that thrive in moist, organic-rich environments (1). *Prototheca bovis* is a causative agent of bovine mastitis, which is often refractory to treatment and can lead to substantial economic losses in the dairy industry worldwide (2). Although a draft genome sequence of *P. bovis* SAG2021 (accession number GCA_003612995.1) is available, genomic information of this pathogen remains limited. In 2024, *P. bovis* strain P18 was isolated from milk samples collected from cattle in Hokkaido Island, Japan, using a previously described method (3). The purified cells were suspended in Cellbanker 1 (Takara Bio Inc., Shiga, Japan) and stored at ‒80  °C until further analysis. This study presents the draft genome sequence of this strain as a reference for Japanese *P. bovis* isolates.

*P. bovis* strain P18 was cultured on Sabouraud dextrose agar plates (BBL, Sparks, MD) at 37°C for 48 h. The cells were harvested and ground into a fine powder in liquid nitrogen using a mortar and pestle. High-molecular-weight genomic DNA (≥10 kb) was purified using the Genomic-tip 100 /G kit (QIAGEN, Hilden, Germany) according to the manufacturer’s instructions. DNA quality and quantity were assessed using a Quantus fluorometer (Promega, Madison, WI). A DNA library was prepared using the SMRTbell Prep Kit 3.0 (PacBio, Menlo Park, CA). Whole-genome sequencing was performed on the PacBio Revio platform with the Revio Polymerase Kit (PacBio) for pooling the SMRTbell library. Circular consensus sequencing (CCS) reads were generated using SMRT Link v13.1.0.221970 (Pacific Biosciences) after the overhang adapter sequences were removed from the raw subreads. CCS reads with a quality value of at least 20 were retained and designated as high-fidelity (HiFi) reads. A total of 155,814 raw reads were generated, with an average read length of 7,755 bp and N50 of 8,935 bp.

Bioinformatic analyses were conducted as described below, using default parameters unless otherwise specified. *De novo* assembly of HiFi reads was performed using Canu v2.2 (4) with the option “genomeSize = 30 m,” followed by extraction of organellar genomes using GetOrganelle v1.7.7.1 (5) with the “embplant_pt” and “embplant_mt” databases. To verify the circularization of the organellar genome, self-BLAST analysis was performed using BLASTN v2.16.0 (6), with the same contig sequences used as both the query and the subject. The tentative circular sequences were mapped and validated using Minimap v0.2-r124 (7) with HiFi reads. The start positions of the detected circular organellar genomes were determined based on the mitochondrion and plastid sequences of *P. bovis* SAG2021. Organellar genome annotation using GeSeq v2.03 (8) was performed with the *P. bovis* SAG2021 mitochondrion and plastid sequences as references. After removing organellar reads from the HiFi data set, *de novo* assembly of the chromosomal genome was conducted using HiFiasm v0.24.0 (9). A total of 27 contigs were assembled for the chromosomal sequences. Telomere identification was performed using tidk v0.2.63 (10). Among these, seven contigs contained telomeric sequences at both ends and 14 contigs had a telomeric sequence at one end, suggesting that *P. bovis* strain P18 possesses 14 chromosomes. Approximately 14% of the chromosomal bases were identified as repetitive regions and were masked using RepeatModeler v2.0.6 (11). Genome annotation was performed using GALBA v1.0.11 (12) with the following reference genome assemblies from unicellular algae: GCA_000690575.1, GCA_002245835.2, GCA_019044685.2, GCA_031763795.1, GCF_000090985.2, GCF_000147415.1, GCF_000258705.1, and GCF_000733215.1. Average nucleotide identity (ANI) between strain P18 and *P. bovis* SAG2021 was determined to be more than 99.4% using fastANI v1.34 (13). Detailed genomic features are summarized in Table 1.

**TABLE 1:** Sequencing statistics and genomic characteristics of *P. bovis* strain P18

Parameters	Genomic features
Organella	
Average size of contigs (bp)	108,017.6
N50 (bp)	257,152
Mitochondrion	
Status	Complete
Nucleotide accession number	AP038925
Contig length (bp)	39,222
GC content (%)	28.71%
Number of CDSs	34
Number of rRNAs	3
Number of tRNAs	17
Mean depth	3,547
Plastid	
Status	Complete
Nucleotide accession number	AP038924
Contig length (bp)	28,830
GC content (%)	26.81%
Number of CDSs	19
Number of rRNAs	3
Number of tRNAs	21
Mean depth	5,446
Chromosome	
Maximum length (bp)	2,902,968
N50 (bp)	1,579,411
Status	Draft
Nucleotide accession number	BAAGFD010000001 - BAAGFD010000027
Number of contigs	27
Total contig length (bp)	27,242,944
GC content (%)	73.58%
Number of CDSs	4,725
Number of rRNAs	144
Number of tRNAs	122
Mean depth	4～146

## Data Availability

The genome sequence of *P. bovis* strain P18 has been deposited in the DDBJ under the following accession numbers: AP038925 (mitochondrion), AP038924 (plastid), and BAAGFD010000001–BAAGFD010000027 (chromosome). The raw PacBio HiFi reads are available at the NCBI Sequence Read Archive (SRA) and can be accessed under the accession number DRR626684. The associated BioProject and BioSample accession numbers are PRJDB19860 and SAMD00856295, respectively.
